# Italian peninsula as a hybridization zone of *Ixodes inopinatus* and *I. ricinus* and the prevalence of tick-borne pathogens in *I. inopinatus*, *I. ricinus*, and their hybrids

**DOI:** 10.1186/s13071-024-06271-z

**Published:** 2024-04-29

**Authors:** Ondřej Daněk, Alena Hrbatová, Karolina Volfová, Sylvie Ševčíková, Paulina Lesiczka, Markéta Nováková, Sajjad Ghodrati, Kristyna Hrazdilova, Vincenzo Veneziano, Ettore Napoli, Domenico Otranto, Fabrizio Montarsi, Andrei Daniel Mihalca, Noureddine Mechouk, Peter Adamík, David Modrý, Ludek Zurek

**Affiliations:** 1 Biology Centre, Institute of Parasitology, Czech Academy of Sciences, České Budějovice, Czech Republic; 2 Department of Veterinary Sciences, Faculty of Agrobiology, Food and Natural Resources, Czech University of Life Sciences, Prague, Czech Republic; 3 CEITEC University of Veterinary Sciences, Brno, Czech Republic; 4 Department of Parasitology, Faculty of Science, Charles University, Prague, Czech Republic; 5 Department of Microbiology, Nutrition and Dietetics, Czech University of Life Sciences, Prague, Czech Republic; 6 Department of Botany and Zoology, Faculty of Science, Masaryk University, Brno, Czech Republic; 7 Faculty of Medicine in Pilsen, Biomedical Center, Charles University, Pilsen, Czech Republic; 8 Department of Veterinary Medicine and Animal Productions, University of Naples Federico II, Naples, Italy; 9 Department of Veterinary Sciences, University of Messina, Messina, Italy; 10 Department of Veterinary Medicine, University of Bari, Valenzano, Italy; 11 Department of Veterinary Clinical Sciences, City University of Hong Kong, Hong Kong, Hong Kong; 12 Instituto Zooprofilattico Sperimentale delle Venezie, Legnaro, Italy; 13 Department of Parasitology and Parasitic Diseases, University of Agricultural Sciences and Veterinary Medicine of Cluj-Napoca, Cluj-Napoca, Romania; 14 Department of Zoology, Palacky University Olomouc, Olomouc, Czech Republic

**Keywords:** *Ixodes inopinatus*, *Ixodes ricinus*, Hybrids, Italy, Algeria, *Borrelia burgdorferi* s.l., *B. miyamotoi*, *Anaplasma phagocytophilum*, *Rickettsia* SFG

## Abstract

**Background:**

*Ixodes inopinatus* was described from Spain on the basis of morphology and partial sequencing of 16S ribosomal DNA. However, several studies suggested that morphological differences between *I. inopinatus* and *Ixodes ricinus* are minimal and that 16S rDNA lacks the power to distinguish the two species. Furthermore, nuclear and mitochondrial markers indicated evidence of hybridization between *I. inopinatus* and *I. ricinus*. In this study, we tested our hypothesis on tick dispersal from North Africa to Southern Europe and determined the prevalence of selected tick-borne pathogens (TBPs) in *I. inopinatus*, *I. ricinus*, and their hybrids.

**Methods:**

Ticks were collected in Italy and Algeria by flagging, identified by sequencing of partial *TROSPA* and *COI* genes, and screened for *Borrelia burgdorferi* s.l., *B. miyamotoi*, *Rickettsia spp.*, and *Anaplasma phagocytophilum* by polymerase chain reaction and sequencing of specific markers.

**Results:**

Out of the 380 ticks, in Italy, 92 were *I. ricinus*, 3 were *I. inopinatus*, and 136 were hybrids of the two species. All 149 ticks from Algeria were *I. inopinatus*. Overall, 60% of ticks were positive for at least one TBP. *Borrelia burgdorferi* s.l. was detected in 19.5% of ticks, and it was significantly more prevalent in *Ixodes* ticks from Algeria than in ticks from Italy. Prevalence of *Rickettsia* spotted fever group (SFG) was 51.1%, with significantly greater prevalence in ticks from Algeria than in ticks from Italy. *Borrelia miyamotoi* and *A. phagocytophilum* were detected in low prevalence (0.9% and 5.2%, respectively) and only in ticks from Italy.

**Conclusions:**

This study indicates that *I. inopinatus* is a dominant species in Algeria, while *I. ricinus* and hybrids were common in Italy. The higher prevalence of *B. burgdorferi* s.l. and *Rickettsia* SFG in *I. inopinatus* compared with that in *I. ricinus* might be due to geographical and ecological differences between these two tick species. The role of *I. inopinatus* in the epidemiology of TBPs needs further investigation in the Mediterranean Basin.

**Graphical Abstract:**

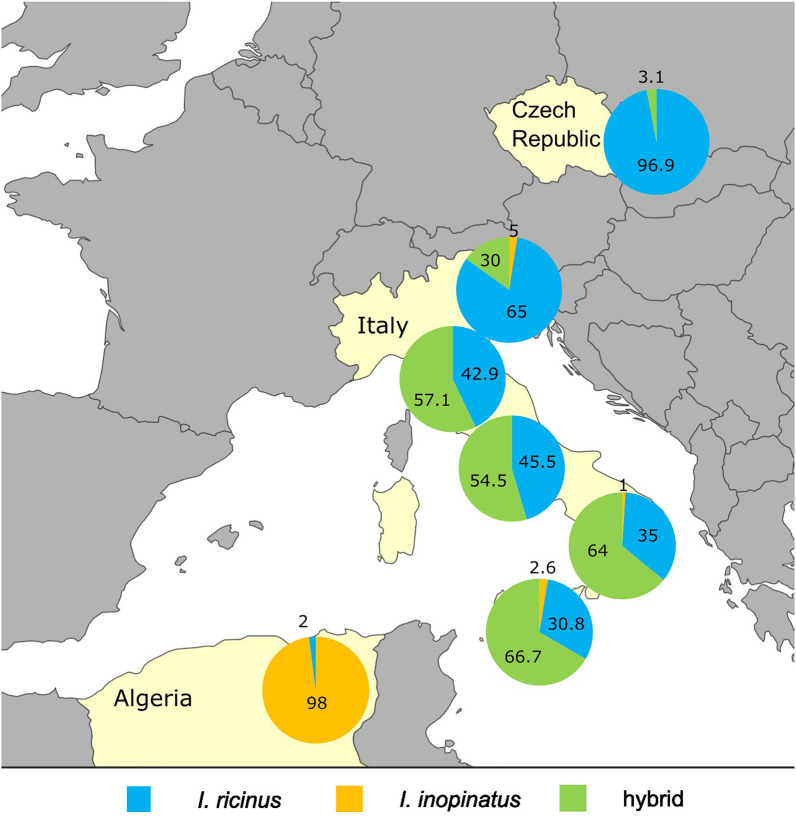

**Supplementary Information:**

The online version contains supplementary material available at 10.1186/s13071-024-06271-z.

## Background

Early research into the genetic structure of the tick *Ixodes ricinus* (Linnaeus, 1758) showed low genetic diversity among European populations; however, it hinted at the existence of a distinct population in North Africa [1, 2]. These findings led in 2014 to the definition of a new species *Ixodes inopinatus* (Estrada-Peña, Nava and Petney, [3]), originally described in Spain and later found in Algeria, Morocco, Tunisia, Portugal, and Germany with the description mainly based on morphology and partial sequencing of the 16S ribosomal RNA (rRNA) gene [3]. *Ixodes inopinatus* was later also reported from Austria, Romania, Turkey, and Tunisia with repeated detections in Germany [4–6]. It has been reported that the main host species of *I. inopinatus* include lizards and foxes [3], although immature stages have been found on migratory birds as well [7].

However, several studies have shown that morphological differentiation of *I. ricinus* and *I. inopinatus* is difficult to unreliable [8, 9], which has led to the use of the term “*Ixodes ricinus*/*inopinatus* complex” in several studies [10, 11]. Furthermore, while 16S rDNA is the genetic marker of choice for delineation of other species of the genus *Ixodes*, recent studies have shown that it is not the appropriate gene target for identifying *I. inopinatus* [12–15]. Different genetic markers such as the nuclear gene for tick receptor *OspA* (*TROSPA*), defensin, internal transcribed spacer 2 (*ITS2*), and mitochondrial cytochrome c oxidase I (*COI*) provided more robust results when compared with tick morphology and 16S rDNA [2, 14]. In our previous study, a combination of nuclear and mitochondrial markers was used to assess the differences between *I. inopinatus* from Algeria and *I. ricinus* from the Czech Republic, with several specimens showing signs of hybridization between these two species [14]. This study, together with a recent study from Germany, questioned all observations of *I. inopinatus* outside the Mediterranean Basin based solely on morphology and/or 16S rDNA [13, 14].

The vector capacity of *I. inopinatus* is unknown, as the majority of studies focusing on tick-borne pathogens in this species used unreliable methods for tick species identification [5, 16], with *Borrelia lusitaniae* being the only detected TBP in reliably identified *I. inopinatus* [17]. However, based on the published data, *I. inopinatus* seems to be a dominant species of genus *Ixodes* in North Africa [2, 14], suggesting that TBPs previously detected in ticks identified as *I. ricinus* were actually at least to some extent detected from *I. inopinatus* [18].

This study is a continuation of our previous study [14] where we suggested that migratory birds are carriers of *I. inopinatus* and/or *I. ricinus*/*I. inopinatus* hybrids into the Czech Republic. Southern Italy is on the route of many migratory birds from northern Africa and Middle East, which often carry different tick species [19] resulting in a large biodiversity of tick species in that area [20, 21].

The aims of this study were to screen flagged ixodid ticks throughout Italy and in Algeria for presence of *I. inopinatus*, *I. ricinus*, and their hybrids, documenting the spread of *I. inopinatus* northward to Central and Northern Europe. Additionally, we screened the collected ticks for four important groups of TBPs, focusing on differences between tick species and their origin.

## Methods

### Tick collection

**Fig. 1:**
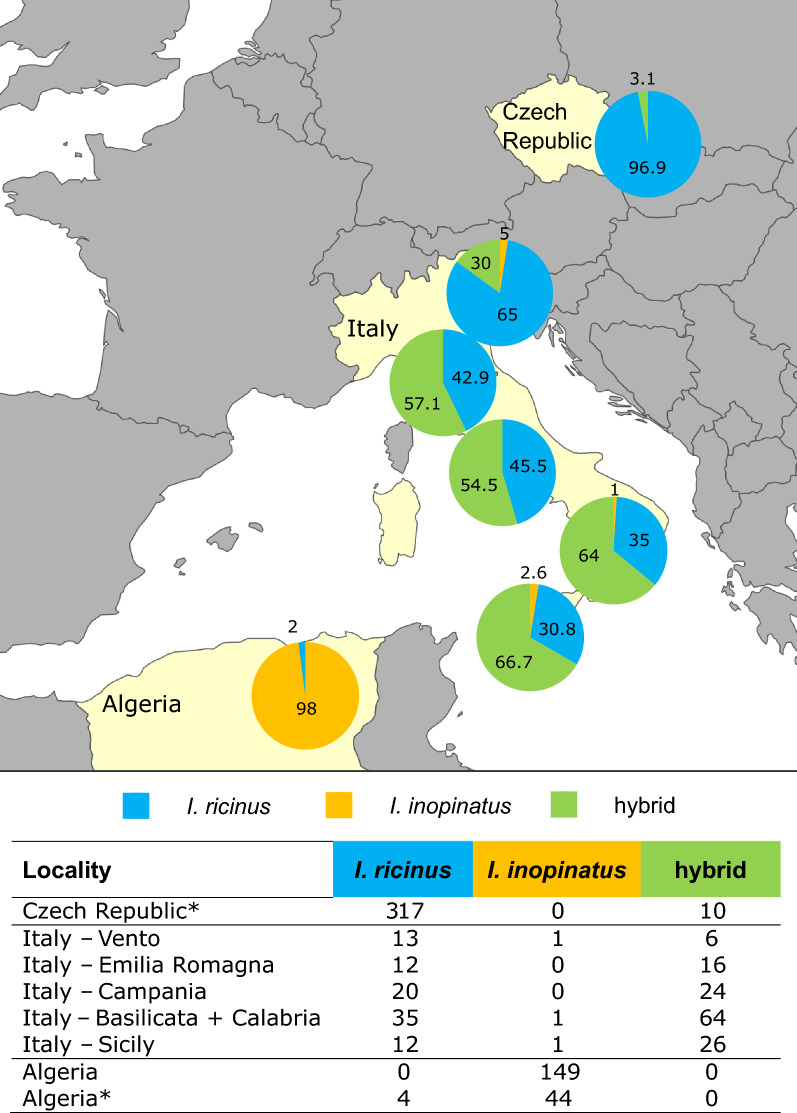
Distribution of *I. ricinus* (blue), *I. inopinatus* (orange), or their hybrids (green) in the Czech Republic, across Italy (from north to south: Veneto, Emilia-Romagna, Campania, Calabria/Basilicata, and Sicily) and in Algeria. Ticks from two localities in Algeria (Oran and El Tarf) and two regions in Italy (Basilicata and Calabria) are presented together in single pie chart, respectively, due to the low number of ticks from Oran (*N* = 5) and Calabria (*N* = 6). Adult ticks from Czech Republic and Algeria classified in a previous study by the same methodology [14] were added to the map (marked with an asterisk in table)

**Fig. 2:**
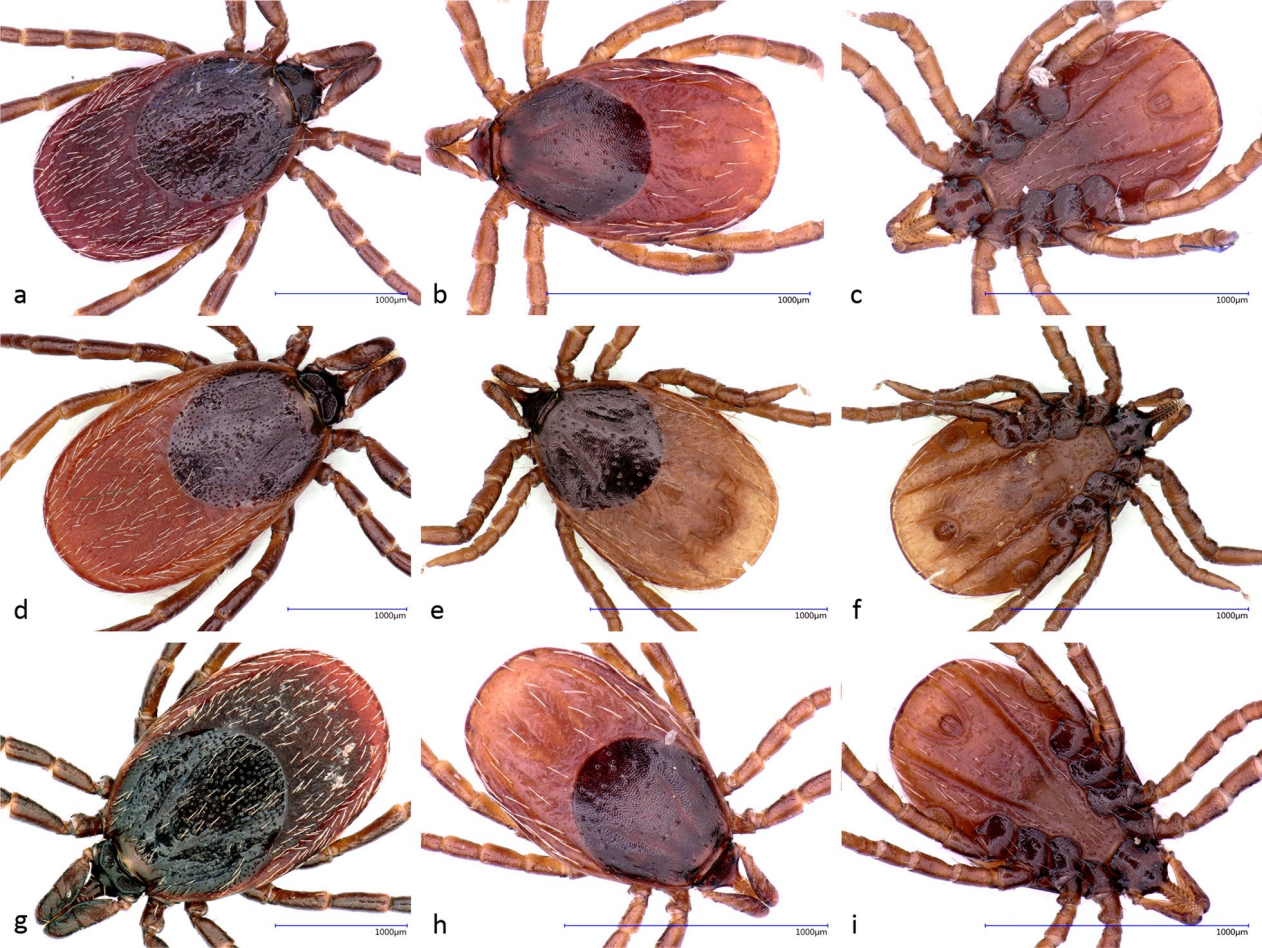
Examples of examined ticks: *Ixodes ricinus* (upper row, **a**–**c**), *Ixodes inopinatus* (**d**–**f**) and *I. ricinus*/*inopinatus* hybrids (**g**–**i**), showing adult aspect of female (right), dorsal (middle), and ventral (right) side of a nymph; depicted *I. ricinus* and hybrids are from Basilicata, Italy, and *I. inopinatus* from El Tarf, Algeria

Ticks were collected by flagging in Italy in spring 2021 and in Algeria in spring 2022. Ticks from Italy were collected from 11 localities in 6 different regions spanning from south to north of the country (Fig. 1 and Additional file 1: Table S1). The majority of ticks form Algeria were flagged in El Tarf Province (northeast Algeria). In each site, up to four transects 250 m each were sampled by flagging using a white cloth 1.0 × 0.5 m attached to a wooden rod, stopping every 5–10 m to check the flag and collect attached ticks [22]. If this initial sampling did not reveal presence of *Ixodes* spp., the locality was not surveyed further (data not shown). Selection of transects was based either on preexisting knowledge on the occurrence of *Ixodes* spp. or based on similarity of altitude and vegetation cover to sites with known occurrence of *Ixodes* spp. in given region. In addition, five specimens were collected partially engorged from animals (four from sheep, one from lizard) in Oran (northwest Algeria) (Additional file 1: Table S1). *Ixodes ricinus*/*I. inopinatus* were not found in Sardinia, despite our flagging efforts comparable to that in other Italian localities. All ticks were stored in 70% ethanol and kept at −20 °C before processing. Ticks were sorted into genera on the basis of general morphology [23], specimens identified as *Ixodes* spp. were photographed (KEYENCE VHX-5000 digital microscope, Keyence, Belgium) (Fig. 2), and those identified as *I. ricinus*/*I. inopinatus* were further examined.

### DNA extraction and tick identification

Genomic DNA was isolated from a longitudinal half of the adult tick or entire nymph using Exgene Cell SV Mini 250p Kit (GeneAll Biotechnology Co. Ltd., South Korea) according to the standard protocol for animal tissues with the following modifications: sterile plastic micropestles were used for thorough homogenization of tick body; homogenized tissue samples with Proteinase K were incubated overnight at 56 °C; 100 µl of elution buffer Buffer EB (Qiagen, Germany) was added in the final step, and the elution step was repeated with the same eluate.

For tick identification, PCR amplification, preparation for sequencing, and sequencing were followed exactly as described in our previous study [14]. To differentiate *I. ricinus* and *I. inopinatus* and their hybrids, sequencing of *TROSPA* and *COI* was conducted. Ticks were identified as *I. ricinus* or *I. inopinatus* if both *TROSPA* and *COI* indicated as such. All specimens with *TROSPA* and *COI* sequences not corresponding to a single species or with double-peak pattern in 23 determining positions in *TROSPA* described by Hrazdilová et al. [14] were assigned as *I. ricinus*/*I. inopinatus* hybrids. Details on primers and protocols are listed in Additional file 1: Table S2.

### Detection of tick-borne pathogens

The presence of four selected TBPs was assessed using either conventional PCR [i.e., *B. burgdorferi* s.l., *Rickettsia* of the spotted fever group (SFG)], qPCR (i.e., *B. miyamotoi*), or nested PCR (samples positive for *B. miyamotoi* on qPCR, *A. phagocytophilum*). For the detection of *B. burgdorferi* s.l., PCR assay targeting the 5S–23S intergenic spacer (IGS) was used. Ticks were tested for the presence of *B. miyamotoi* using a qPCR protocol targeting the *glpQ* gene, and all positive samples were subjected to nested PCR targeting the same gene. The presence of *Rickettsia* SFG was tested by two PCR assays. The first targeted partial sequence of the *gltA* gene of 401 bp represented the genus *Rickettsia* [24]. The positive samples were subsequently tested with the second assay targeting 632 bp sequence of the *ompA* gene. These primers are specific for *Rickettsia* SFG except for *R. helvetica* [25, 26]. The detection of *A. phagocytophilum* was based on nested PCR targeting a fragment of the *groEL* operon (1297 bp) or (in the case of a missing amplicon) of the *groEL* gene (407 bp) as previously described [27]. Subsequently, the samples were analyzed using two nested PCR assays targeting the *ankA* gene. The primers and details on all PCR assays are listed in Additional file 1: Table S2.

Amplicons were separated by electrophoresis in a 1.5% agarose gel stained with Midori Green Advance (Nippon Genetics Europe, Germany) and visualized under ultraviolet (UV) light. The PCR products of the expected size were either excised from the gel and purified using the Gel/PCR DNA Fragments Kit (Geneaid, Taiwan) or cleaned by the ExoSAP-IT^™^ PCR Product Cleanup Reagent (Applied Biosystems^™^, USA). Purified products were sequenced in both directions using the amplification primers by the Sanger technology. Sequence analysis was performed by SeqMe (Czech Republic) or by Macrogen Capillary Electrophoresis Sequencing services (Macrogen Europe, the Netherlands). The sequences obtained were processed using the Geneious Prime^®^ software version 2022.0.1 [28] and compared with those available in the GenBank^®^ dataset by the Basic Local Alignment Search Tool (BLAST).

### Phylogenetic analysis

To assess the zoonotic potential of *A. phagocytophilum*, we conducted phylogenetic analysis. For *groEL*, phylogenetic analysis was performed on 143 sequences from the GenBank representing all four described ecotypes [21] and eight clusters [22] together with 7 unique sequences from this study and sequences of *A. platys* as an outgroup. The *ankA* gene phylogenetic analysis was performed on 74 sequences from the GenBank representing different clusters together with 14 unique sequences from this study and sequences of *A. marginale* as an outgroup. Due to an uneven length of sequences, the alignments were calculated in two steps by the MAFFT algorithm, using “Auto” strategy for sequences > 1000 nt and “add” function for implementing shorter sequences.

### Statistical analysis

To detect statistically significant difference in prevalence of tick-borne pathogens between tick species, Pearson’s chi-squared test with Yates’ correction for cases with two samples was used. For expected frequencies below 5, Fisher’s exact test (FET) was used. All analyses were done in R version 4.3.2.

## Results

### Tick identification

Based on morphology, 380 ticks (231 from Italy and 149 from Algeria) were identified as *I. ricinus*/*I. inopinatus*. Additional nine ticks (six *I. gibbosus* and three *I. frontalis*; confirmed by 16S rDNA and *TROSPA* [14]) were found in Italy and excluded from this study. Based on the molecular analysis, in Italy, 92 ticks were identified as *I. ricinus*, 3 as *I. inopinatus*, and the remaining 136 were hybrids of these two species (95 based on double peaks in *TROSPA* and the remaining 41 ticks based on the discrepancy between *TROSPA* and *COI*) (Table 1). All 149 ticks collected in Algeria were identified as *I. inopinatus* on the basis of the combination of *TROSPA* and *COI* (Table 1). Regarding the *COI*, similarity of obtained sequences with sequence of *I. ricinus* (GU074892) ranged from 98.99% to 100%. Concerning the TROSPA, sequences belonging to the “*ricinus* variant” of the gene showed 98.65–99.77% similarity to the sequence of *I. ricinus* (GU074852), and sequences identified as the “*inopinatus* variant” of the gene showed 98.25–100% similarity to the sequence of *I. inopinatus* (HM461042). The sequences with double peaks in the 23 determining positions [14] showed lower similarity to the available sequences ranging from 96.5% to 97.86% in *I. inopinatus* (HM461042) and 96.97–97.9% in *I. ricinus* (GU074852). The geographic distribution of collected ticks combined with our previous results from the Czech Republic [14] is shown in Fig. 1. Representative sequences were deposited in the NCBI GenBank database with accession numbers in Additional file 1: Table S3.

**Table 1:** Identification of ticks from Italy (ITA) and Algeria (ALG) based on sequencing of *TROSPA* and *COI* gene fragments

*TROSPA*	*COI*	*TROSPA* + *COI*
ITA		
95* I. ricinus*	92 *I. ricinus*	
	**3* I. inopinatus***	
32 *I. inopinatus*	**29* I. ricinus***	92 *I. ricinus*
	3 *I. inopinatus*	3 *I. inopinatus*
104 hybrids	**98 *I. ricinus***	**136 hybrids**
	**6* I. inopinatus***	
ALG		
0 *I. ricinus*		
149 *I. inopinatus*	0* I. ricinus*	149 *I. inopinatus*
	149 *I. inopinatus*	
0 hybrid		

Ticks classified as hybrids between *I. ricinus* and *I. inopinatus* on the basis of *TROSPA* and *COI* sequences are marked in bold

### Prevalence of tick-borne pathogens

Results of TBPs detected in screened ticks (*n* = 380) are reported in Tables 2 and 3, and representative sequences of pathogen haplotypes were deposited in the NCBI GenBank database (Additional file 1: Table S3). *Borrelia burgdorferi* s.l. was detected in 19.5% (*n* = 74) ticks. Based on the tick origin, *B. burgdorferi* s.l. was significantly more prevalent in Algeria (36.9%, 55/149) than in Italy (8.2%, 19/231) (*χ*^2^ = 42.0, *P* < 0.0001, Table 2). Regarding the tick species, *B. burgdorferi* s.l. was more frequently found in *I. inopinatus* (37.5%, 57/152) than in *I. ricinus* or hybrids of both species (Table 3). Altogether, eight unique *Borrelia* haplotypes were detected. Based on BLAST analysis, seven haplotypes were *B. lusitaniae* (73 ticks, 98.4–99.7% sequence identity) and one haplotype was *B. valaisiana* (one tick). *Borrelia miyamotoi* was found in 0.9% (2/231) ticks and only in Italy (Tables 2, 3). Both ticks positive for *B. miyamotoi* were hybrid nymphs, and their sequences were identical to several sequences in GenBank (e.g., MK256773).

**Table 2:** Prevalence of tick-borne pathogens (TBPs), *B. burgdorferi* s.l., *B. miyamotoi*, *Rickettsia* spp., and *A. phagocytophilum*, in ticks from Italy (ITA) and Algeria (ALG)

	Tick origin (total number of ticks)		
	ITA (231)	ALG (149)	
	TBPs prevalence % (number of positive *N*/*F*/*M*)		*P* value
*Borrelia burgdorferi* s.l.	8.2 (15/2/2)	36.9 (44/5/6)	< 0.00001*
*B. lusitaniae*	7.8 (14/2/2)	36.9 (44/5/6)	< 0.00001*
*B. valaisiana*	0.4 (1/0/0)	0 (0/0/0)	1.0
*Borrelia miyamotoi*	0.9 (2/0/0)	0 (0/0/0)	0.523
*Rickettsia* spp.	31.2 (42/23/7)	81.9 (99/15/8)	< 0.00001*
*R. helvetica*	6.5 (10/4/1)	22.2 (29/1/3)	0.0002
*R. monacensis*	24.7 (32/19/6)	59.7 (70/14/5)	< 0.00001*
*A. phagocytophilum*	5.2 (8/3/1)	0 (0/0/0)	0.004*

Prevalence of given TBP in tested ticks (%) followed by number of TBP-positive nymphs (*N*), adult females (*F*), and adult males (*M*)

*Statistical significance at *P* < 0.05

**Table 3:** Prevalence of tick-borne pathogens (TBPs), *B. burgdorferi* s.l., *B. miyamotoi*, *Rickettsia* spp., and *A. phagocytophilum*, in *I. ricinus*, *I. inopinatus*, and their hybrids originating from Italy (ITA) and Algeria (ALG)

	Assignment based on *TROSPA* and *COI* sequencing (total number of ticks)			
	*I. ricinus* (92)	*I. inopinatus* (152)	Hybrids (136)	
	TBP prevalence % (number of positive *N*/*F*/*M*)			*P* value
*Borrelia burgdorferi* s.l.	7.6 (7/0/0)	37.5 (ITA 2/0/0, ALG 44/5/6)	7.4 (6/2/2)	< 0.00001*
*B. lusitaniae*	6.5 (6/0/0)	37.5 (ITA 2/0/0, ALG 44/5/6)	7.4 (6/2/2)	< 0.00001*
*B. valaisiana*	1.1 (1/0/0)	0 (0/0/0)	0 (0/0/0)	0.244
*Borrelia miyamotoi*	0 (0/0/0)	0 (0/0/0)	1.5 (2/0/0)	0.187
*Rickettsia* spp.	28.3 (14/8/4)	80.9 (ITA 1/0/0, ALG 99/15/8)	33.1 (27/15/3)	< 0.00001*
*R. helvetica*	7.6 (4/2/1)	21.7 (ITA 0/0/0, ALG 29/1/3)	5.9 (6/2/0)	< 0.00001*
*R. monacensis*	20.7 (10/6/3)	59.2 (ITA 1/0/0, ALG 70/14/5)	27.2 (21/13/3)	< 0.00001*
*A. phagocytophilum*	2.2 (2/0/0)	0 (0/0/0)	7.4 (6/3/1)	0.00088

Prevalence of given TBP in tested ticks (%) followed by number of TBP-positive nymphs (*N*), adult females (*F*), and adult males (*M*)

*Statistical significance at *P* < 0.05

The overall prevalence of *Rickettsia* SFG was 51.1% (194/380), with significantly higher prevalence in ticks from Algeria (81.9%, 122/149) than from Italy (31.2%, 72/231) (*P* < 0.0001, Table 2). *Rickettsia* SFG were more frequently detected in *I. inopinatus* (80.9%, 123/152) than in either *I. ricinus* (28.3%, 26/92) or hybrids (33.1%, 45/136) (Table 3). Based on the BLAST analysis of partial sequences of *gltA* and *ompA*, all *Rickettsia* SFG were either *R. helvetica* (12.6%, 48/380) or *R. monacensis* (38.4%, 146/380) (Tables 2, 3). Only one haplotype of *gltA* was detected in both *R. helvetica* and *R. monacensis* and three *ompA* haplotypes (haplotypes A, B, and C) in *R. monacensis*. The type A was most common in Algeria and rare in Italy (found in five ticks: four *I. ricinus* and one *I. inopinatus*), and the type B was present in most tested ticks in Italy and only in one nymph of *I. inopinatus* in Algeria. The type C was found in a single nymph of *I. inopinatus* from Algeria.

*Anaplasma phagocytophilum* was detected only in ticks from Italy (5.2%, 12/231). Based on tick species *A. phagocytophilum* was more frequently found in hybrids (7.4%, 10/136) than in *I. ricinus* (2.2%, 2/92), and no *I. inopinatus* was positive for this pathogen (Tables 2 and 3). Both protocols of nested PCR targeting fragments of *groEL* yielded amplicons of expected size in all 12 ticks. Among all sequences, we identified seven unique genetic variants with 99.35–99.91% sequence identity. The analysis targeting the *ankA* gene was also successful in all 12 ticks, with PCR yielding products of the expected size. Among all sequences, we identified 11 unique genetic variants with 62.6–99.5% sequence identity (Additional file 1: Table S4).

Overall, 60% (*n* = 228) of ticks in this study were positive for at least one of the selected TBPs, with co-infections detected in 25.4% (*n* = 58) of positive ticks. All co-infected ticks were positive for *Rickettsia* SFG and one other pathogen with most frequent co-infections with *B. burgdorferi* s. l. (86.2%, 50/58) followed by *A. phagocytophilum* (12.1%, 7/58) and *B. miyamotoi* (1.7%, 1/58).

Based on the classification of *A. phagocytophilum* [29, 30], three well-supported phylogenetic clades of partial *groEL* sequences were distinguished (Additional file 2: Fig. S1). The largest clade consists of two ecotypes, I and II. Eleven isolates from our study belonged to ecotype I together with isolates from ungulates, dogs, horses, and *Ixodes* spp. The second well-distinguishable clade consists of isolates of ecotype III with clusters V and VI. The last clade is represented by a small number of sequences in ecotype IV/cluster VII. In our study, one sample belonged to ecotype IV, and none of them clustered in ecotypes II and III.

Our phylogenetic analysis of the *ankA* gene following [31] revealed three well-supported clades (Additional file 2: Fig. S2). The first clade consists of six clusters of which cluster 1 is the most diverse and contains all of the sequences isolated from humans from Europe and sequences from small, medium, and large mammals and *I. ricinus*. Nine of our sequences were placed in this cluster 1. The second well-supported clade consists of five clusters. Four of the sequences obtained in this study belonged to cluster 4, which consists of sequences from wild and domestic ruminants and *I. ricinus* from Europe. Four of our sequences belong to cluster 4. The third and smallest clade contains only a single cluster 6 with sequences from birds and *Ixodes* spp. from birds; one sequence from our study belonged in this clade.

## Discussion

Since the description of *I. inopinatus* in 2014 from the Iberian Peninsula and North Africa based on morphological differences from *I. ricinus* and the partial sequence of 16S rDNA [3], this species has been reported from several European countries, with the highest number of reports from Germany [4, 5, 10]. However, recent studies showed that previously used methods of differentiation of *I. inopinatus* from closely related *I. ricinus* are unreliable, and prior findings outside the Mediterranean Basin relying on 16S rDNA and/or morphology alone should be reexamined [13, 14]. Based on the molecular data other than 16S rDNA, *I. inopinatus* is a dominant *Ixodes* sp. in North Africa [2, 15] and is also present on the Iberian Peninsula [17] and in Italy [7]. It has been demonstrated experimentally that hybridization of closely related species *I. ricinus*, *I. persulcatus*, and *I. pavlovskyi* can occur [32, 33]. We previously found signs of hybridization between *I. inopinatus* and *I. ricinus* using a combination of nuclear (*TROSPA*) and mitochondrial (*COI*) markers, showing a need to use more than one marker to characterize individuals of the *I. ricinus*/*inopinatus* complex [14]. By analyzing available data and our sequences, we have found that *I. inopinatus* and several possible *I. ricinus*/*inopinatus* hybrids were reported also from Turkey (based on *defensin*) [34], and three *I. inopinatus* or *I. ricinus*/*inopinatus* hybrids were unknowingly reported from Croatia (*COI*; MZ305534), Poland (*COI*; OP882707), and Russia (*TROSPA*; KU669029), respectively [35–37].

We hypothesized in our previous study [14] that migratory birds are responsible for transport of *I. ricinus*/*inopinatus* hybrids from Mediterranean Basin to the Czech Republic and other countries in Central Europe. In the current study, we aimed to assess the population of the *I. ricinus*/*inopinatus* complex in Italy, which is one of the first important “stopover region” between North Africa and Central Europe for many migratory birds [7, 14]. In striking difference to our previous data from the Czech Republic, most ticks from Italy were *I. ricinus*/*inopinatus* hybrids, based on analysis of partial sequences of *TROSPA* and *COI*. While mitochondrial markers such as *COI* cannot reveal direct signs of hybridization, they can be used to detect hybridization when used in combination with nuclear markers. In our study, several ticks with the sequence of *TROSPA* clearly corresponded to *I. inopinatus*, while the *COI* sequence corresponded to *I. ricinus* and vice versa, identifying them as hybrids. Interestingly, in hybrids, the *COI* variant corresponding to *I. ricinus* vastly outnumbered the *I. inopinatus* variant of this gene. This was also the case of hybrids from the Czech Republic [14]. This phenomenon could indicate differences in viability and/or reproductive capabilities of hybrids from different parent tick species, favoring the offspring of female *I. ricinus* and male *I. inopinatus*. A similar phenomenon has been reported for hybrids of *Dermacentor variabilis* and *D. andersoni* in North America [38]. Nevertheless, subsequent hybridization studies are necessary to address this phenomenon.

In contrast to our data from Italy, all ticks collected in Algeria in 2022 were *I. inopinatus*. As we reported several adult *I. ricinus* from Algeria [14], it is likely that *I. ricinus* and *I. inopinatus* are sympatric in this region; however, *I. inopinatus* appears to be dominant. While both species are present in this region, no signs of hybridization in North Africa have been observed so far. This could be because of low prevalence and survival rate of *I. ricinus* due to hotter and drier climate, to which *I. inopinatus* is adapted. Another explanation might be due to differences in seasonality of developmental stages of ticks in relation to direction of bird migration. Nonetheless, most ticks in both studies were collected from the same region in eastern Algeria, and additional data are needed to assess the prevalence and distribution of *I. ricinus* and hybrids in North Africa using reliable methodologies.

Birds are known as hosts of juvenile *Ixodid* ticks, and migratory species can transport them over long distances, including between continents [7, 19]. *Ixodes inopinatus* was found previously on migratory birds on the Island of Ventotene [7]. While only three ticks were identified as *I. inopinatus*, authors were able to identify less than 39% of *Ixodes* spp. using 16S rDNA confirmed by *TROSPA*. This means that larvae and nymphs of *I. inopinatus* might be much more prevalent on migratory birds flying from North Africa to Europe. This hypothesis is also supported by the fact that, in our study, the percentage of detected hybrids was highest in Sicily and southern Italy and decreased northward. We propose that juvenile stages of *I. inopinatus* are transported by migratory birds and likely drop off at the first resting stop of these birds. There, the surviving adults of *I. inopinatus* hybridize with *I. ricinus* and contribute to the dominant population of hybrids of both species. Furthermore, migratory birds can, in turn, carry hybrids further north. As previous studies relied on 16S rDNA and/or morphology or were conducted before the description of *I. inopinatus*, they were unable to detect the hybrids. We suggest that the continuous spread of *I. inopinatus* northward has likely been happening for a long time, and *I. ricinus*/*inopinatus* hybrids might be a dominant ixodid tick in some regions of southern Europe, especially along major migration corridors of birds.

Based on the available data, with the exception of one report on *Borrelia lusitaniae* from *I. inopinatus* based on *TROSPA* sequences [17], there are no reports on prevalence of TBP in this species and nothing is known on pathogens carried by *I. ricinus*/*inopinatus* hybrids. Nowadays, 23 different *Borrelia* genospecies are considered to constitute the complex of *B. burgdorferi* s.l. [39], 10 of which were detected in humans, suggesting their potential to cause clinical symptoms of Lyme disease [40]. In our study, we detected two *Borrelia* genospecies *B. lusitaniae* and *B. valaisiana*, both previously isolated from humans. Reptiles, especially lizards, serve as the main reservoir hosts for *B. lusitaniae*, the genospecies widely spread in southern Europe and North Africa [e.g., 17, 18, 41–43]. *Borrelia* *lusitaniae* has been also reported from Central Europe (Slovakia, Czech Republic, Poland) and Latvia [44–47]. Furthermore, the recent work of Norte et al. [17] using MLST (Multi-locus sequence typing) analysis showed that *B. lusitaniae* forms two populations: one from Mediterranean Basin and the other from the rest of Europe, including northern Portugal [17]. These authors hypothesized that this population division is due to *Borrelia* host association, considering both lizard and tick species. While the sequences obtained in our study were relatively uniform with one SNP (Single nucleotide polymorphism) separating sequences from Algeria and Italy, we cannot compare our results to the above-mentioned study as our marker (IGS) was not part of the used MLST (Multi-locus sequence typing) analysis [17]. In our study, the prevalence of *B. lusitaniae* was much higher in Algeria than in Italy, which is in accordance with previous studies [18, 48]. Interestingly, the two *I. inopinatus* found in Italy were positive for *B. lusitaniae*. These data suggest a higher affinity of *B. lusitaniae* to *I. inopinatus* and/or show preference of immature *I. inopinatus* stages to feed on lizards. The observed prevalence in hybrids was slightly higher than that in *I. ricinus*, showing potential difference in ecology or vector capacity of the hybrids. Clearly, further studies are needed to assess the hybrids for their role in eco-epidemiology of TBDs. *Borrelia valaisiana*, a bird-associated species, has been sporadically reported from Italy previously [49–51], and this is supported in our study as only one tick was positive for *B. valaisiana*. *Borrelia afzelii* and *B. garinii*, species frequently detected in Italy [43, 50, 52], were not found in our study. This could be due to a limited number of ticks from northern Italy, where both *B. afzelii* and *B. garinii* were previously detected.

*Borrelia miyamotoi* is an emerging TBP belonging to the relapsing fever group of borreliae. So far, it has been detected in Europe, Asia, and North America [53]. In Europe, *B. miyamotoi* is transmitted by *I. ricinus*, and *I. persulcatus*, with wild rodents serving as reservoir hosts [54, 55]. This TBP has been reported in low prevalence from northern Italy previously [56, 57], which is in correlation to our data, as only two ticks (hybrids) were positive for *B. miyamotoi* in this study, and both originated from northern Italy.

The genus *Rickettsia* is traditionally divided into the spotted fever group, the typhus group, the *R. bellii*, and the *R. canadensis* groups, with most of the tick-borne rickettsioses caused by the SGF [58]. Rickettsiae were the most prevalent TBPs detected in this study. We found *R. helvetica* and *R. monacensis*, both belonging to the SFG [59, 60]. The prevalence of both *R. monacensis* and *R. helvetica* were significantly higher in ticks from Algeria than in ticks from Italy, and with *R. monacensis* as dominant species in both countries. While both species were detected previously in North Africa, data from larger datasets of questing ticks are missing [61–63]. Our data from Italy are in concordance with the recent study from southern Italy [64]. The prevalence of *Rickettsia* SFG in questing *I. ricinus* collected in various parts of Europe differs significantly; however, the prevalence is usually low [52, 65–68], with the exception of studies conducted in large cities where it can reach above 50% [69, 70]. Regarding the detected species, *R. helvetica* is the most prevalent *Rickettsia* detected in *I. ricinus* in the above studies. In vertebrates, *R. helvetica* has been reported from rodents and forest habitats [66] and from lizards in dry environments [64, 71].

The host preference of *Rickettsia* SFG and differences in a host spectrum can explain the dominance of *R. monacensis* in this study, since lizards are likely main hosts of larval ticks in North Africa and southern Italy. The striking difference in prevalence between North Africa and Italy is likely due to multiple factors, mainly the environment, the vertebrate host, and the tick vector. Another explanation could be a higher survival rate of *Rickettsia* SFG in *I. inopinatus* compared with that of *I. ricinus*. However, more studies are necessary to make any conclusions as most ticks from Algeria were collected in a relatively small area. When analyzing the obtained sequences of *ompA* of *R. monacensis*, two major haplotypes (differing by 5 SNP/~ 550 bp) almost precisely corresponding to geographic origin of the tick were detected (“A” in Algeria and “B” in Italy). These could be variants adapted to different vertebrate host groups or even adapted to either *I. ricinus* or *I. inopinatus*; however, this needs to be studied further.

*Anaplasma phagocytophilum* is a widespread TBP responsible for clinical disease in humans and domestic animals, with *I. ricinus* as the main vector in Europe [72, 73]. The low prevalence of *A. phagocytophilum* detected in this study is in correlation with previous studies conducted on questing *I. ricinus* in Italy [74–76]. While the mentioned studies were conducted mostly in northern Italy, all ticks positive for *A. phagocytophilum* in our study originated from southern Italy, where this TBP was detected in different hosts [77, 78]. The prevalence of *A. phagocytophilum* in hybrids was much higher than that in *I. ricinus*. This could be due to the fact that almost two-thirds of ticks in this region were hybrids rather than due to the vector preference. Interestingly, all ticks from North Africa were negative for this pathogen. Previously, *A. phagocytophilum* was reported from Algeria in cattle with clinical disease [79], and several studies detected this pathogen in neighboring countries in ticks of the *I. ricinus*/*inopinatus* complex [80, 81]. The absence of *A. phagocytophilum* in our samples might be due to the host preference of *I. inopinatus*. While lizards are a poor hosts of *A. phagocytophilum*, they are likely preferred by immature stages of *I. inopinatus*, and most of the north African ticks in this study were nymphs [3, 82].

Several genotypes of *A. phagocytophilum* are described on the basis of *groEL* and *ankA* phylogenies, with preferred host groups for different genotypes [29–31]. While *A. phagocytophilum* is regarded as zoonotic pathogen, all sequences isolated from human cases in Europe belong to ecotype I (*groEL*)/cluster 1 (*ankA*) [27]. The majority of sequences obtained in this study belonged to zoonotic cluster 1 of ecotype I based on *groEL*; however, several of these were part of cluster 4 based on *ankA*. Nonetheless, 10 of 12 ticks positive for *A. phagocytophilum* in this study harbored potentially zoonotic genotypes of this pathogen. This is in correlation to a reported case of human granulocytic anaplasmosis (HGA) in Sicily [83] and shows potential for emergence of this disease in southern Italy.

## Conclusions

This study shows that *I. inopinatus* is a dominant tick species in Algeria, while southern Italy is likely the hybridization zone of *I. ricinus* and *I. inopinatus*. The extent of observed hybridization in Italy suggests that small numbers of hybrids are continuously carried northward by migratory birds, which explains their presence in the Czech Republic and Central Europe in general. As the mitochondrial *COI* sequences show dominance of *I. ricinus* mitochondrial DNA among the tested hybrids, further studies should focus on hybridization of both species, specifically the viability of offspring of different species pairs in F1 and subsequent generations. This is vital for understanding the ecology and potential spread of *I. inopinatus* and hybrids to Europe. This study contributes important data on TBPs in credibly identified *I. inopinatus*. The prevalence of *B. burgdorferi* s.l. and *Rickettsia* SFG is much higher in *I. inopinatus* than that in *I. ricinus*, and this might be due to geographical and ecological differences between the tick species rather than their different vector capacity. The high prevalence of reptile-associated *B. lusitaniae* and *R. monacensis* in *I. inopinatus* show the host preference of this tick species. While the prevalence of *Borrelia burgdorferi* s.l. and *Rickettsia* SFG in this study was relatively high, the detected species are rarely associated with disease in humans [59, 84, 85]. Further studies focusing on the distribution and vector capacity of *I. inopinatus* and *I. ricinus*/*I. inopinatus* hybrids are needed to assess its role in the epidemiology of TBPs in the Mediterranean Basin and beyond.

### Supplementary Information

**Additional file 1: Table S1.** Details on tick collection. **Table S2.** Details of PCR protocols. **Table S3.** Accession numbers of sequences submitted to the GenBank. **Table S4.** *Anaplasma phagocytophilum* genotypes.

**Additional file 2: Figure S1.** *A. phagocytophilum* *groEL* phylogeny. **Figure S2.** *A. phagocytophilum* *ankA* phylogeny.

## Data Availability

The nucleotide sequences generated in the present study have been deposited in GenBank (https://www.ncbi.nlm.nih.gov/), with detailed information present in Additional file 1: Table S3.
